# Exploring the Role of Obesity in Dilated Cardiomyopathy Based on Bio-informatics Analysis

**DOI:** 10.3390/jcdd9120462

**Published:** 2022-12-15

**Authors:** Xuehua Wang, Wei Liu, Huili Li, Jiaxing Ding, Yu Feng, Zhijian Chen

**Affiliations:** 1 Department of Cardiology, Union Hospital, Tongji Medical College, Huazhong University of Science and Technology, Wuhan 430022, China; 2 Hubei Key Laboratory of Biological Targeted Therapy, Union Hospital, Tongji Medical College, Huazhong University of Science and Technology, Wuhan 430022, China; 3 Hubei Provincial Engineering Research Center of Immunological Diagnosis and Therapy for Cardiovascular Diseases, Union Hospital, Tongji Medical College, Huazhong University of Science and Technology, Wuhan 430022, China; 4 Department of Cardiology, Wuhan Fourth Hospital, Wuhan 430033, China

**Keywords:** dilated cardiomyopathy, obesity, bio-informatics analysis, GEO, LASSO

## Abstract

(1) Background: Obesity is a major risk factor for cardiovascular disease (CVD), contributing to increasing global disease burdens. Apart from heart failure, coronary artery disease, and arrhythmia, recent research has found that obesity also elevates the risk of dilated cardiomyopathy (DCM). The main purpose of this study was to investigate the underlying biological role of obesity in increasing the risk of DCM. (2) Methods: The datasets GSE120895, GSE19303, and GSE2508 were downloaded from the Gene Expression Omnibus (GEO) database. Differentially expressed genes (DEGs) were analyzed using GSE120895 for DCM and GSE2508 for obesity, and the findings were compiled to discover the common genes. Gene Ontology (GO) and Kyoto Encyclopedia of Genes and Genomes (KEGG) pathway enrichment analyses were conducted for the common genes in RStudio. In addition, CIBERSORT was used to obtain the immune cellular composition from DEGs. The key genes were identified in the set of common genes by the least absolute shrinkage and selection operator (LASSO) algorithm, the prognostic risk models of which were verified by receiver operator characteristic (ROC) curves in GSE19303. Finally, Spearman’s correlation was used to explore the connections between key genes and immune cells. (3) Results: GO and KEGG pathway enrichment analyses showed that the main enriched terms of the common genes were transforming growth factor-beta (TGF-β), fibrillar collagen, NADPH oxidase activity, and multiple hormone-related signaling pathways. Both obesity and DCM had a disordered immune environment, especially obesity. The key genes *NOX4*, *CCDC80*, *COL1A2*, *HTRA1*, and *KLHL29* may be primarily responsible for the changes. Spearman’s correlation analysis performed for key genes and immune cells indicated that *KLHL29* closely correlated to T cells and M2 macrophages, and *HTRA1 very tightly* correlated to plasma cells. (4) Conclusions: Bio-informatics analyses performed for DCM and obesity in our study suggested that obesity disturbed the immune micro-environment, promoted oxidative stress, and increased myocardial fibrosis, resulting in ventricular remodeling and an increased risk of DCM. The key genes *KLHL29* and *HTRA1* may play critical roles in obesity-related DCM.

## 1. Introduction

Dilated cardiomyopathy (DCM) is a disease of the myocardium with left ventricular (LV) dilation and systolic dysfunction resulting from genetic and environmental factors, excepting coronary artery disease and abnormal loading [1]. DCM, a major cause of heart failure, is the most common indication for heart transplantation worldwide, the prevalence of which is about 40 in 100,000 [2]. Genetic factors play important roles in the development of DCM. In addition, infection, auto-immune diseases, drugs, toxins, metabolism, and endocrine disturbances have also been shown to participate in DCM occurrence [2]. With the co-action of genetic and nongenetic factors in DCM, cardiomyocytes become necrotic and develop fibrotic patches and calcification. This leads to increased diastolic pressure and ventricular dilatation, further resulting in a decreased left ventricular ejection fraction (LVEF) and typical signs of heart failure [3]. The prognosis of DCM is poor with a 5-year survival rate of less than 50% [4]. Obesity, a metabolic disease defined by a BMI (body mass index) ≥ 30 kg/m^2^, shows close association with cardiovascular disease (CVD), including heart failure, coronary artery disease, and arrhythmia [5]. Nearly 2 billion adults worldwide are overweight (BMI 25.0–30.0 kg/m^2^) or obese, strongly increasing all-cause mortality, most of which can be attributed to CVD [6,7]. The growing rate of childhood obesity is also related to the increased prevalence of cardiovascular morbidity and mortality [8]. Both obesity and DCM are more common in patients less than 55 years of age than in older patients [9]. However, there are no pharmaceutical interventions targeting obesity specifically to cut down the morbidity and mortality of CVD [10].

Little attention has been paid to the association between DCM and obesity over the past few decades: some have suggested that there was little association between them, while several recent studies have suggested the contrary [11,12]. Robertson and his team spent 46 years following up on a prospective cohort study of almost 1.7 million Swedish adolescent men and found that increasing BMI was closely tied to an elevated risk of cardiomyopathy, especially DCM [13]. The same was found to hold true for young women [14]. In patients with DCM, obesity led to a need for increased stroke volume, which was achieved by excessive LV cavity dilatation, which exacerbated ventricular remodelling [15]. Animal models showed that fatty acids (FA) and glucose, serving as the primary fuel for the heart, as well as tetralinoleoyl cardiolipin (CL), were found to be decreased in DCM and obesity as seen in [16]. However, the pathophysiological link between the two is not quite clear and we are trying to further understand it by using bio-informatics methods.

In the era of big data, the field of bio-informatics is developing at a great speed. Bio-informatics is used to acquire, store, organize, archive, analyze, and visualize biological data [17]. Bio-informatics plays an essential role in modern biology and medicine for data management, enabling research to shift focus from single-gene pathways to cellular networks of genes, and to identify their role in disease [18]. Precision medicine, a new therapeutic concept and method, has become a new star on the stage, with the development of bio-informatics, big data, and omics showing enormous potential in CVD [19].

In our study, we have used bio-informatics methods to explore the underlying link between obesity and DCM, which could be helpful in the treatment of DCM patients with obesity.

## 2. Materials and Methods

### 2.1. Micro-Array Data

Gene expression profiles of DCM (GSE120895 [20], GSE19303 [21]) on platform GPL570 and gene expression profiles of obesity (GSE2508 [22]) on platform GPL92 were downloaded from the Gene Expression Omnibus (GEO) database (http://www.ncbi.nlm.nih.gov/geo/, accessed on 13 May 2022) and logarithmically transformed in RStudio. Samples of DCM and control samples were all taken from human endocardium myocardium. GSE120895 was used as the training dataset, and included 47 DCM patients and 8 controls. GSE19303, which was used to validate the key genes, contained 40 human endocardium myocardium samples from DCM at baseline, and 8 control samples, of which 33 DCM samples were obtained again six months later, after immune-adsorption with subsequent immunoglobulin substitution (IgA/IgG). The 33 patients with symptoms of HF caused by DCM did not develop cancer, infectious diseases, coronary heart disease, acute myocarditis, or other conditions that lead to HF [21]. GSE2508 contained 39 samples of adipocytes from 19 obese subjects and 20 lean individuals.

### 2.2. Identification of Differentially Expressed Genes (DEGs)

Differentially expressed genes (DEGs) for both datasets were identified by RStudio with package “limma” [23], filtered by |Log2 fold change| > mean + (twice the standard deviation), and *p*-value < 0.05. Up- and down-regulated genes were shown in volcano plots. The common genes were mapped using the Draw Venn Diagram web tool (http://bioinformatics.psb.ugent.be/webtools/Venn/, accessed on 25 May 2022).

### 2.3. Identification of Genes Related to Ferroptosis and Immune Response

We obtained a total of 388 ferroptosis genes from FerrDb [24] (http://www.zhounan.org/ferrdb/current/, accessed on 25 May 2022) and 1793 immune-related genes from the Immunology Database and Analysis Portal [25] (ImmPort, https://www.immport.org/home, accessed on 25 May 2022), separately intersecting with common genes from the Draw Venn Diagram web tool.

### 2.4. Function Enrichment Analysis of Common Genes

Gene Ontology (GO) and Kyoto Encyclopedia of Genes and Genomes (KEGG) pathway enrichment analyses were conducted for the common genes in RStudio with package “clusterProfiler” [26], setting *p*-value < 0.05. Gene Ontology (GO) described the biological functions of common genes at biological process (BP), cellular component (CC), and molecular function (MF) levels.

### 2.5. CIBERSORT

CIBERSORT is a method for obtaining immune cellular composition from gene expression profiles using a deconvolution algorithm and leukocyte gene signature matrix LM22 [27]. For the analysis of the immune micro-environment for DCM and obesity, CIBERSORT was performed on DEGs with 1000 permutations in RStudio. The results were visualized by the “ggplot2” package.

### 2.6. Identification and Verification of Key Genes

The least absolute shrinkage and selection operator (LASSO) algorithm was used to identify the key genes among the common genes with R package “glmnet” [28]. Subsequently, lambda.min and lambda.1se (standard error, SE) were selected to construct the prognostic risk models in GSE120895 and GSE2508, respectively, which were then verified by receiver operator characteristic (ROC) curves in GSE19303. Gene expression profiles of 15 common genes from GSE19303 were used to verify the values of the models to distinguish between DCM and control, and to assess the ability to discriminate obese and lean in the DCM group. Genes from the intersection of the models (with lambda.min) of GSE120895 and GSE2508 were identified as key genes. In order to further evaluate the diagnosis value of key genes, we drew ROC curves and calculated the area under the curve (AUC) for each gene in SPSS.

### 2.7. Spearman’s Correlation Analysis between Key Genes and Infiltrating Immune Cells

Spearman’s correlation analysis was performed on the obese DCM set using the R packages “psych” and “ggcorrplot”, to explore the connections between the key genes and immune cells, with 0.05 as a *p*-value cutoff.

## 3. Results

### 3.1. Identification of Common Genes

**Figure 1:**
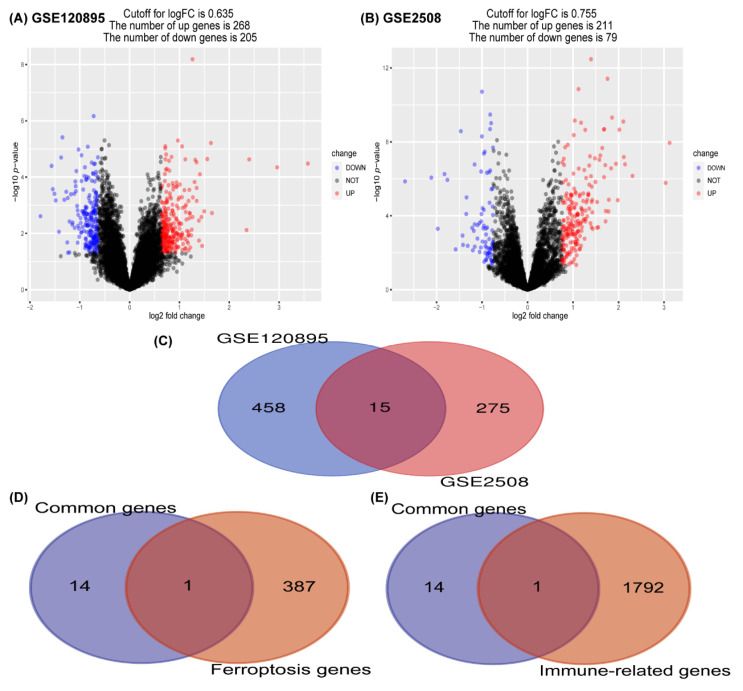
Identification of common genes in GSE120895 and GSE2508. (**A**) The volcano plot shows differentially expressed genes (DEGs) in GSE120895. Red indicates up-regulated genes, and blue indicates down-regulated genes. (**B**) The volcano plot shows DEGs in GSE2508. (**C**) Venn diagram of DEGs in two datasets. (**D**) Venn diagram of common genes and ferroptosis genes. (**E**) Venn diagram of common genes and immune-related genes.

A total of 473 DEGs belonging to GSE120895 were visualized in a volcano plot (Figure 1A), including 268 up-regulated and 205 down-regulated genes. Additionally, 290 differentially expressed genes (DEGs) were identified in GSE2508 (Figure 1B), of which 212 were up-regulated and 78 were down-regulated. The intersection of these differentially expressed gene (DEG) collections had 15 genes (Figure 1C), including *NOX4*, *MBD6*, *PPP2R2C*, *PDE3B*, *ADAMTS15*, *TPPP3*, *MXRA5*, *CCDC80*, *PHLDA1*, *PRSS23*, *COL1A2*, *HTRA1*, *CREB5*, *KLHL29*, and *ASPN. MBD6*, *PPP2R2C,* and *ADAMTS16* were down-regulated, while the others were up-regulated in DCM. However, there were some differences in DCM from obesity—*MBD6*, *CREB5,* and *PDE3B* were down-regulated and the others were up-regulated. To summarize, among the common genes, *MBD6* was the only gene down-regulated in both datasets; *PPP2R2C*, *ADAMTS16*, *CREB5* and *PDE3B* had varied regulation; and the rest of the genes were up-regulated.

In addition, we separately intersected common genes with immune genes and ferroptosis genes, and found that *NOX4* was the only common gene in both intersections (Figure 1D,E).

### 3.2. Analysis of Common Genes at Functional Level

**Figure 2:**
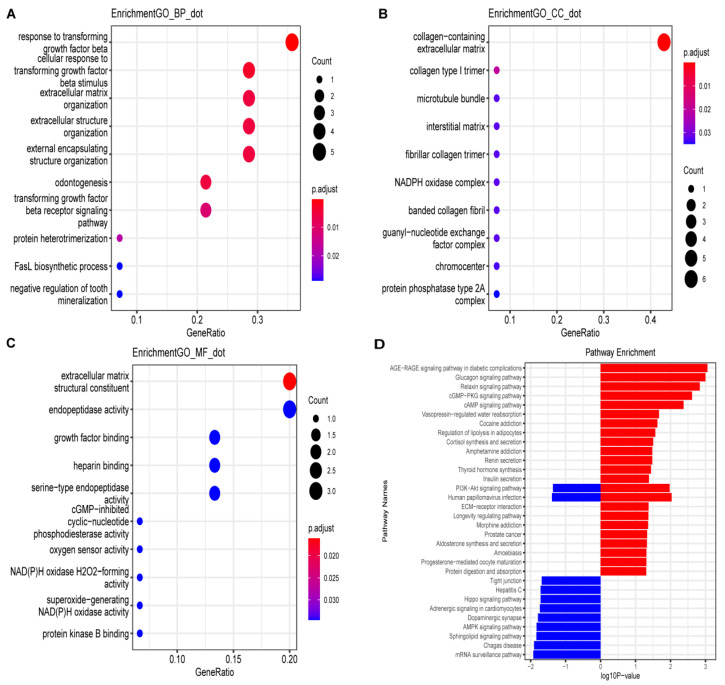
Gene ontology (GO) and Kyoto Encyclopedia of Genes and Genomes (KEGG) pathway enrichment analyses of the 15 common genes. Dot sizes represent counts of enriched genes, and dot colors represent the adjusted *p* value. P.adj < 0.05. (**A**) GO terms involved by common genes in biological process (BP) category. (**B**) GO terms in cellular component (CC) category. (**C**) GO terms in molecular function (MF) category. (**D**) KEGG pathway enrichment analysis of 15 common genes.

Gene Ontology (GO) and Kyoto Encyclopedia of Genes and Genomes (KEGG) pathway enrichment analyses were performed for 15 common genes to decipher biological functions in DCM. Gene Ontology (GO) enrichment analysis showed that the main enriched terms in the biological process (BP) category included the response to transforming growth factor-beta (TGF-β), several kinds of extracellular organization, and the FasL biosynthetic process (Figure 2A). The collagen-containing extracellular matrix, fibrillar collagen and NADPH oxidase complex were enriched in the cellular component (CC) category (Figure 2B). Extracellular matrix structural constituents, growth factor binding, heparin binding, and the activity of several enzymes (endopeptidase, NADPH oxidase, phosphodiesterase) were the major enriched terms in the molecular function (MF) category (Figure 2C). According to Kyoto Encyclopedia of Genes and Genomes (KEGG) analysis, the terms mainly enriched were the AGE-RAGE signaling pathway in diabetic complications, cGMP-PKG, cAMP and PI3K-Akt signaling pathways, human papillomavirus infection, regulation of lipolysis in adipocytes, and multiple hormone (relaxin, aldosterone, vasopressin, adrenergic, thyroid hormone, insulin, glucagon)-related signaling pathways (Figure 2D). This showed that the endocrine system plays an important role in DCM.

### 3.3. Immune Cell Infiltration

**Figure 3:**
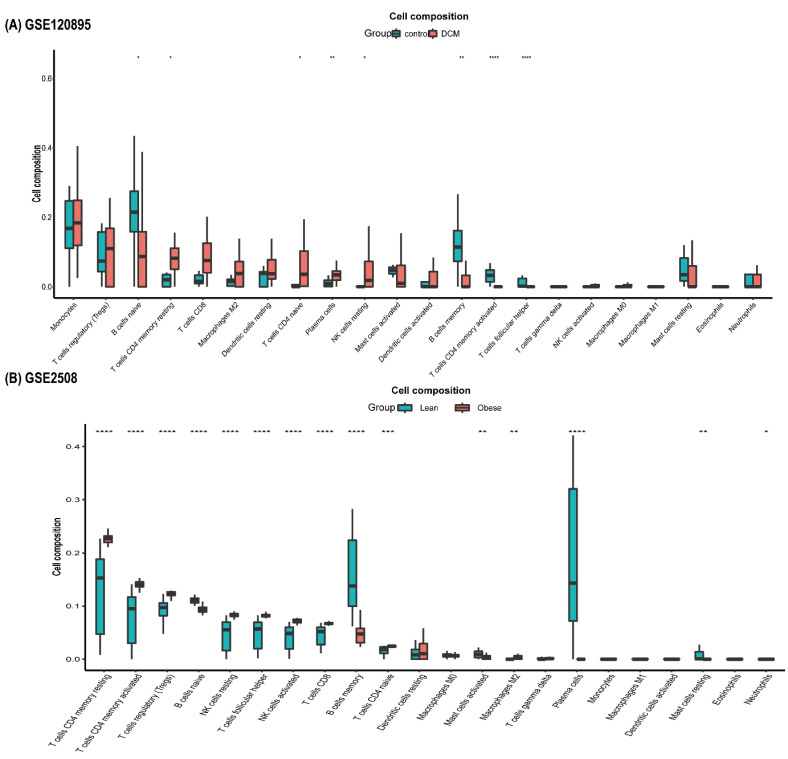
The boxplot of immune cells. (**A**) The boxplot of immune cells in GSE120895. (**B**) The boxplot of immune cells in GSE2508. * *p* < 0.05, ** *p* < 0.01, *** *p* < 0.001, **** *p* < 0.0001.

Both DCM and obesity showed differences from control in immune cell infiltration (Figure 3). The immune environment of obesity was more disordered, where most kinds of immune cells had a dysregulation. Compared to control, both DCM and obesity groups had a higher proportion of resting memory CD4 and naive T cells, as well as resting NK cells. The number of naive and memory B cells in DCM and obesity was less than that in control.

### 3.4. Identification and Verification of Key Genes

**Figure 4:**
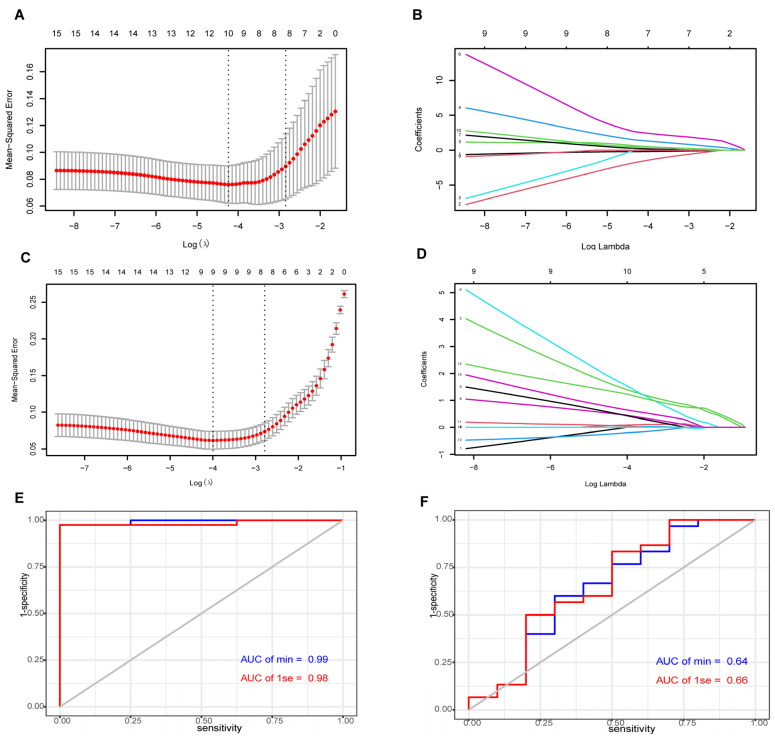
LASSO regression analysis and model discrimination. (**A**) Selection of lambda minimum and 1-SE criteria in the LASSO regression analysis in GSE120895. (**B**) LASSO coefficient profiles of common genes in GSE120895. (**C**) Selection of lambda minimum and 1-SE criteria in the LASSO regression analysis in GSE2508. (**D**) LASSO coefficient profiles of common genes in GSE2508. (**E**) ROC curves for assessing the value of models to diagnose DCM in GSE19303. (**F**) ROC curves for model verification in DCM from GSE19303, grouped by BMI.

Key genes were further identified from common genes by LASSO in the training datasets (Figure 4). Lambda.min and lambda.1se (standard error, SE) were 0.014 and 0.058 (Figure 4A), which were used to construct LASSO regression models in GSE120895. Lambda.min and lambda.1se of LASSO regression models in the GSE2508 datasets were 0.018 and 0.062 (Figure 4C). *NOX4*, *MBD6*, *PDE3B*, *ADAMTS15*, *TPPP3*, *CCDC80*, *PHLDA1*, *COL1A2*, *HTRA*, and *KLHL29* in the LASSO model with lambda.min were selected in GSE120895, while the model with lambda.min in GSE2508 missed *MBD6*, *ADAMTS15*, *PHLDA1*, *TPPP3*, and *PDE3B.* Therefore, *NOX4 (nicotinamide adenine dinucleotide phosphate oxidase 4)*, *CCDC80 (coiled-coil domain-containing 80)*, *COL1A2 (collagen type I alpha 2 chain)*, *HTRA1 (the high temperature requirement factor A1),* and *KLHL29 (Kelch-like family member 29)* were identified as key genes.

ROC curves further verified the value of the two models to make a distinction between DCM and control in the validation datasets, the AUCs of which were 0.99 and 0.98 (Figure 4E), respectively. However, the differences between lean and obese DCM were not obvious, with both AUCs being <0.7 (Figure 4F).

**Figure 5:**
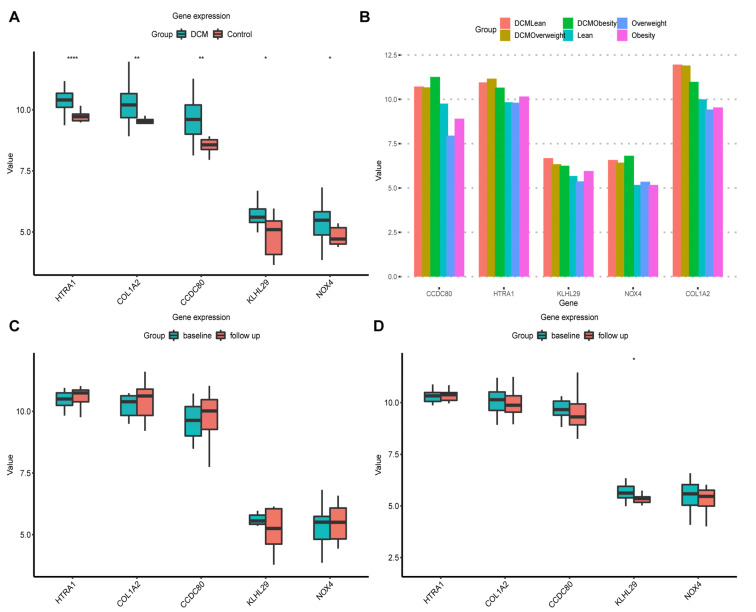
GSE19303 was used to verify the expression levels of key genes in each group. (**A**) The boxplot of key gene expression in DCM and control group. (**B**) The baseline samples in GSE19303 divided into six groups by DCM and BMI. (**C**) The boxplot of key gene expression before and after IgA/IgG in 13 nonresponders. (**D**) The boxplot of key gene expression before and after IgA/IgG in 20 responders. * *p* < 0.05, ** *p* < 0.01, **** *p* < 0.0001.

The five key genes were all obviously up-regulated in DCM (Figure 5A,B), the AUCs of which were calculated in GSE19303 (Table 1). We chose 40 DCM samples at different BMI baseline levels and four lean control samples to perform ROC analysis and calculate the AUCs (Table 2), indicating that the key genes had significant diagnostic value for DCM. Compared to lean or overweight DCM, the AUCs of *CCDC80*, *NOX4*, and *COL1A2* dropped a lot in obese DCM and the control group, while *HTRA1* (AUC = 0.9, *p* = 0.024) and *KLHL29* (AUC = 0.8, *p* = 0.066) showed stable values within normal limits. A total of 33 patients were followed up after IgA/IgG, including 20 responders and 13 non-responders. Only *KLHL29* had a statistically significant decrease in responders (Figure 5C,D).

### 3.5. Spearman Correlation Analysis between Key Genes and Infiltrating Immune Cells

**Figure 6:**
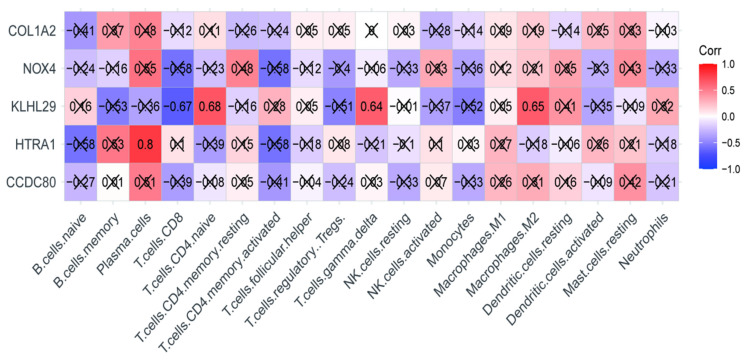
Correlation analysis between five key genes and immune cells. Purple squares represent negative correlation, and red squares represent positive correlation. The correlation coefficient showing in the squares is between −1 and +1, and the larger the absolute value, the stronger the association. × *p* > 0.05.

Spearman correlation analysis was performed for key genes and immune cells (Figure 6), which indicated that *KLHL29* and *HTRA1* strongly correlated to immune cells in DCM. *KLHL29* was closely associated negatively with CD8 T cells (cor = −0.67, *p* < 0.05), and positively with CD4 naive T cells (cor = 0.68, *p* < 0.05) and gamma delta (γδ) T Cells (cor = 0.64, *p* < 0.05). *KLHL29* was also fairly linked with M2 macrophages (cor = 0.65, *p* < 0.05). *HTRA1* strongly correlated to plasma cells (cor = 0.8, *p* < 0.05).

## 4. Discussion

Given the increasing global disease burden of CVD, there is no doubt that obesity as one of its main risk factors contributes to the burden [29]. Heart failure accounts for a majority of the CVD burden, for which DCM is one of the main causes [2]. Although the relationship between DCM and obesity has been explored in literature, and the scientific community acknowledges obesity as a risk factor for DCM, the pathological link is not clear [13,14]. Bio-informatics was used to reveal potential connections between DCM and obesity in this study. Initially, we derived 15 common genes between DCM and obesity via an analysis of DEGs. Enrichment analysis showed that the common genes mainly played a role in TGF-β, fibrillar collagen, NADPH oxidase activity, and multiple hormone-related signaling pathways, which suggested that metabolic disorders, oxidative stress, and myocardial fibrosis might play a role in the obesity-induced development of DCM. In addition, both DCM and obesity had a similar immune micro-environment, possibly involving *NOX4*, related to immune response and ferroptosis. *NOX4*, *CCDC80*, *COL1A2*, *HTRA1*, and *KLHL29* were identified as key genes by LASSO regression analysis, among which *HTRA1* and *KLHL29* showed a close association with immune system infiltration in DCM and obesity.

Obesity exposes the body to chronic inflammation. It impacts immune system function by disrupting the structure and function of lymphoid tissue and altering the distribution of white blood cells [30]. Immune cells are recruited to respond to the myocardial inflammation in DCM, whether from an infection or due to an auto-immune response [31]. Our study revealed that obesity showed a more disturbed immune environment compared to DCM, in which almost all immune cell populations are altered. The increased adipose tissue in obesity may be responsible for the disordered immune environment [32]. We found that M2 macrophages increased in both DCM and obesity, but especially in obesity. Adipose tissue macrophages (ATM) play a central role in obesity-associated inflammation. M2 macrophages are alternatively activated macrophages playing a role in the anti-inflammatory response, and undergo conversion to M1 as obesity progresses [33]. Increased infiltration of M2 macrophages into the myocardium in DCM is independently associated with cardiac fibrosis, leading to a poor prognosis [34]. T-cell infiltration is another typical inflammatory infiltration, which plays a part in the pathogenesis of inflammation in DCM [34]. *HTRA1* and *KLHL29* had a high correlation with CD8 T cells, CD4 naive T cells, gamma delta (γδ) T cells, M2 macrophages, and plasma cells. *HTRA1*, a highly conserved serine protease, is ubiquitous in various organisms, involved in certain signaling pathways, and participating in various disease pathogeneses [35]. *HTRA1* and oxidative stress act synergistically to promote macrophage infiltration and inflammation in age-related macular degeneration (AMD) [36]. Although the role of *HTRA1* in DCM has not been reported, D Colak [37] found *HTRA1* significantly up-regulated (6.9-fold) in DCM and suggested that *HTRA1* may contribute to cardiomyopathy pathways. Another interesting discovery suggested that *HTRA1* was mainly expressed in plasma cells in inflamed gingival tissue [38], echoing our findings that *HTRA1* had a high correlation coefficient to plasma cells (cor = 0.8, *p* < 0.05). It is reasonable to consider that *HTRA1* plays a significant role in the cardiomyopathy inflammation of DCM. *HTRA1* expression is increased in obese patients, especially in insulin-resistant (IR) adipose tissue, possibly related to the developmental and functional deficits of the adipocytes [39]. The levels of *HTRA1*, a negative regulator of mesenchymal stem cell (MSC) adipogenesis, and matrix metalloproteinase-13 (MMP-13) proteins, which played an important role in the pathophysiology of adipose tissue, were evidently high in the visceral adipose tissue of IR obese patients and also in the cardiac tissue of DCM patients [38,39,40], lending support to *HTRA1* mediating the potential pathogenesis of DCM and obesity. *KLHL29* is a protein-coding gene belonging to the conserved *Kelch-like (KLHL)* gene family whose expression is associated with micro-fragmented adipose tissue (MF), exerting an anti-inflammatory effect in osteoarthritis [41]. There is little research on *KLHL29*, but in our study *KLHL29* was the only gene obviously decreased with statistical significance after immunotherapy in responders. Furthermore, *KLHL29* had good AUCs and a close relation to CD8 T cells, CD4 naive T cells, gamma delta (γδ) T cells, and M2 macrophages in our study, which indicated that *KLHL29* had a potential association with obesity and DCM, especially immunologically.

Oxidative stress is significantly associated with obesity and DCM [42,43]. *NOX4* is a kind of isoform of *NOX* whose main biological function is to generate reactive oxygen species (ROS), expressed in various cardiovascular tissues and playing a complex role in the development of CVD [44]. *NOX4* expression increasing in catalase-knockout mice adipocytes resulted in both adipogenesis and lipogenesis [45]. The increasing fatty acids in adipocytes induces the activation of NADPH oxidase (a main enrichment term in our study), increasing oxidative stress and production of ROS, ultimately causing metabolic syndrome [46]. Our results showed that *NOX4* had an elevated expression in both DCM and obesity, connecting with immune response and ferroptosis. Ferroptosis promoted by oxidative stress-induced lipid peroxidation increases myocardial fibrosis, resulting in cardiomyopathy [47]. The activation of nucleotide-binding domain and leucine-rich repeat pyrin domain containing 3 (NLRP3) inflammasomes was closely associated with ferroptosis [48]. *NOX4* may be involved in DCM progression by activating NLRP3 inflammasomes [49]. It is reasonable to assume that obesity promotes the development of DCM via *NOX4* participating in the immune response and ferroptosis by activating NADPH oxidase and NLRP3 inflammasomes. The pathogenesis association needs to be explored via further study.

Intramyocardial fibrillar collagen increased significantly in patients with DCM, especially type I collagen [50]. *COL1A2* encodes the pro-alpha2 chain of type I collagen, which is a main fibrillar collagen produced by heart fibroblasts [51]. TGF-β up-regulates the expression of *COL1A2* in cardiac tissues and vascular smooth muscle, antagonizing the inhibitory effect of interferon gamma (IFN-γ) on it [51,52]. Obesity participates in cardiac fibrosis by up-regulating the expression of *COL1A1* and *COL1A2* in cardiac fibroblasts and further significantly increasing myocardial collagen content [53]. *CCDC80*, a protein secreted by adipocytes that regulates lipogenesis, is significantly elevated in visceral adipose tissue (VAT) in obesity [54]. *CCDC80* has a close association with the fibrillin-1 affected by TGF-β signaling, and may be involved in the regulation of vascular tone [55]. Given that *COL1A2*, closely related to *CCDC80*, mediates the production of fibrillar collagen, we may assume that the combined action of *CCDC80* and *COL1A2* promotes myocardial fibrosis and accelerates the development of cardiomyopathy in the presence of obesity risk factors.

This study revealed the underlying relation between DCM and obesity by bio-informatic analysis, with a focus on oxidative stress, collagen synthesis, immune response, and ferroptosis. Antonini-Canterin et al. developed another index which showed a better performance than BMI in evaluating body fat-related cardiovascular risk, named the waist-corrected BMI (wBMI), calculated as the waist circumference (WC) × BMI [56]. Indeed, numerous studies have shown that BMI is not a good indicator of the risk of obesity comorbidities [57]. Performing a comprehensive clinical evaluation of obesity can provide a deeper understanding of obesity-related DCM. Further experiments and clinical trials are needed to validate our results and explore potential mechanisms of pathophysiology.

## 5. Conclusions

This study based on bio-informatics analyses found that obesity might exacerbate the development of DCM by having a stimulatory effect on collagen synthesis, influenced by immune response and ferroptosis related to oxidative stress. The key genes, *NOX4*, *CCDC80*, *COL1A2*, *HTRA1*, and *KLHL29* were significantly expressed in DCM, with *KLHL29* and *HTRA1* especially closely associated with the immune response, which might be markers for obesity-induced DCM and be potential avenues for exploration in immunotherapy.

## Figures and Tables

**Table 1:** Area under the curves (AUC).

Grouped	Gene	Area	Std. Error ^a^	Asymptotic Sig ^b^	95% CI	
					Lower Bound	Upper Bound
DCM-Lean vs. control	CCDC80	0.827	0.139	0.054	0.554	1.000
	HTRA1	1.000	0.000	0.003	1.000	1.000
	KLHL29	0.827	0.139	0.054	0.554	1.000
	NOX4	0.923	0.069	0.013	0.788	1.000
	COL1A2	0.750	0.121	0.141	0.512	0.988
DCM-Overweight vs. control	CCDC80	0.794	0.110	0.073	0.578	1.000
	HTRA1	0.971	0.036	0.004	0.899	1.000
	KLHL29	0.824	0.120	0.049	0.588	1.000
	NOX4	0.809	0.096	0.060	0.621	0.997
	COL1A2	0.838	0.087	0.039	0.668	1.000
DCM-Obesity vs. control	CCDC80	0.600	0.159	0.572	0.289	0.911
	HTRA1	0.900	0.095	0.024	0.714	1.000
	KLHL29	0.825	0.139	0.066	0.552	1.000
	NOX4	0.575	0.151	0.671	0.278	0.872
	COL1A2	0.575	0.152	0.671	0.277	0.873

^a^ Under the nonparametric assumption. ^b^ Null hypothesis: true area = 0.5.

**Table 2:** Characteristics of individuals in GSE19303.

	DCM Patients (*n* = 33)			Control (*n* = 8)
	Responder (*n* = 20)	Non-Responder (*n* = 13)	All Patients (*n* = 33)	
Age (years) ± SD	48 ± 10	53 ± 8	50 ± 9	43 ± 15
Male sex (%)	70	69	70	75
BMI (kg/m^2^) ± SD	28 ± 5	27 ± 4	28 ± 5	26 ± 5
LVEF (%) ± SD	33 ± 6	35 ± 7	34 ± 6	60 ± 8
LVIDd ± SD	67 ± 7	74 ± 7	70 ± 8	51 ± 3

BMI Body mass index, LVEF left ventricular ejection fraction, LVIDd left ventricular internal diameter at diastole. Mean values with standard deviation (SD) are shown.

## Data Availability

The data presented in this study are openly available in Gene Expression Omnibus (GEO) (URL: https://www.ncbi.nlm.nih.gov/geo/, accessed on 13 May 2022).
